# Supplementary material for: School reopening and risks accelerating the COVID-19 pandemic: A systematic review and meta-analysis protocol
Source: PLoS One. 2021 Nov 17;16(11):e0260189. doi: 10.1371/journal.pone.0260189 (PMC8598030; doi:10.1371/journal.pone.0260189)
Supplement: S1 File — (DOCX) [file pone.0260189.s002.docx]

| ***Study number:*** | | **Methodological Appraisal tool:** |
| --- | --- | --- |
| **STUDY CARACTERISTICS** | | |
| Authors |  | |
| Title |  | |
| Year of publication |  | |
| Country |  | |
| Conflicts of interests |  | |
| Sponsorship |  | |
| Background |  | |
| Rationale |  | |
| Hypothesis tested |  | |
| Objectives |  | |
| ***Methods*** | | |
| Methodology is reported according with STROBE  ( ) Yes  ( ) No  ( ) Partially | | |
| Study design |  | |
| If, cohort study | I. Number of participants in the exposed and unexposed cohort:  II. Number of participants in each group:  III. Comparability of exposed and unexposed cohorts  IV. Contamination (unexposed patient being exposed):  V. Follow-up period:  VI. Dropouts: | |
| If, case-control study | I. Criteria for selection of cases:  II. Criteria for selection of controls:  III. Comparability of groups:  IV. Dropouts: | |
| Local: |  | |
| Sample size and calculation: |  | |
| Inclusion criteria (definition of exposure of interest) |  | |
| Exclusion criteria |  | |
| Confounding factors/Interaction factors considered |  | |
| Ethical aspects |  | |
| Procedure for data collection:  - Collection period:  - Procedures: |  | |
| Instruments for data collection |  | |
| Outcomes / Evaluation of outcomes  - Primary outcome:  - Secondary outcome: |  | |
| Follow-up |  | |
| Statistical analysis |  | |
| ***Results*** | | |
| Main results |  | |
| Clinical-Epidemiological Significance |  | |
| Limitations of the study |  | |
| Strengths of the study |  | |
| ***Conclusions*** | | |
| Main conclusions |  | |
| Implication for clinical practice and research or for decision-makers / stakeholders |  | |

**Supplementary material 1.** Data extraction form based on previous publications [39,41-44].
