# Supplementary material for: School reopening and risks accelerating the COVID-19 pandemic: A systematic review and meta-analysis protocol
Source: PLoS One. 2021 Nov 17;16(11):e0260189. doi: 10.1371/journal.pone.0260189 (PMC8598030; doi:10.1371/journal.pone.0260189)
Supplement: Attachment — Submitted filename: Letter in response to Editors August 10 2021.pdf [file pone.0260189.s003.pdf]

LETTER IN RESPONSE TO EDITORS

Vitória, ES, Brazil, August 10th, 2021

Dear Editorial Board of the *PLoS One*

We would like to re-submit our paper entitled ***"School reopening and risks accelerating the COVID-19 pandemic: a systematic review and meta-analysis protocol"*** by *Lopes-Júnior et al*. to your journal.

Below we have responded in detail point by point the items raised in the previous submission, which have all been addressed.

PONE-D-21-24128
School reopening and risks accelerating the COVID-19 pandemic: a systematic review and meta-analysis protocol
PLOS ONE

Dear Dr. Lopes-Junior,

Thank you for submitting your manuscript for review by PLOS ONE. After careful consideration, we have decided that your manuscript does not meet our publication criteria and therefore must be rejected.

Specifically, during our initial internal evaluation of the manuscript, we found significant text overlap and re-use between your submission and the following previously published work(s) outside the Methods section.

https://www.bmj.com/content/372/bmj.n521
https://science.sciencemag.org/content/372/6546/1092.full
http://jogh.org/documents/issue202002/jogh-10-021104.pdf
https://www.thelancet.com/journals/lancet/article/PIIS0140-6736(21)00622-X/fulltext

PLOS ONE would like to make you aware that copying extracts from previous publications word-for-word is unacceptable. In addition, the reproduction of text from published reports may have implications for any copyright that applies to the publications.

While we understand that some text overlap and re-use may be unavoidable in the Methods section, we require that any overlap is minimized in the Introduction, Results, and Discussion sections. Text from overlapping sources must be cited, quoted, or rephrased as is appropriate.

Can you please write to the journal with comments on this matter?
Response: Thank you so much for your message as well as your valuable and careful/relevant commentaries. First of all, I apologize for this. Actually, I have checked the versions of the article and found that I attached a very preliminary version that I was still working on and also rephrasing. Therefore, the previous article sent [PONE-D-21-24128] was immediately denied.
I sent an email to PLos One who kindly replied and asked me to adapt it for a new submission.
In fact, I got confused when attaching the files and I am so sorry for this mistake. The file that should have been attached is now in the PLOS One manager system for new analysis. In this new version presented, all these issues regarding the significant text overlap and re-use were addressed.

In addition, the quality of the English language in your submission is not at an adequate standard for peer review. We recommend having your manuscript copy edited for language usage and grammar, for instance by a third party or a professional service.
Response: Regarding the quality of the English, as per recommended we have paid for having our manuscript copy edited for language usage and grammar, by a professional service in order to become adequate standard for peer review (we have also uploaded the certificate of this service in the PLOS One manager system).

In addition, we note that you have submitted your study as a Registered Report Protocol. Please note that Registered Report Protocols submitted to PLOS ONE should report the study proposal prior to conducting experiments, data collection or patient recruitment (https://journals.plos.org/plosone/s/other-article-types#loc-registered-reports). In this case, we note that article selection was started on 30 June 2021. Preliminary or pilot data may be included, but only if necessary to support the feasibility of the study or as a proof of principle. As such, this submission does not meet our criteria for registered report protocols.
Response: Absolutely, this is a Registered Report Protocol with PROSPERO/NHS, and we authors claim that we haven't processed the study selection yet. In fact, the only thing that was done, so far, was a pilot of the search strategy in a single database (MEDLINE/PubMed) and that's why we submitted the protocol to be considered for publication in your journal. We do not process the search strategy and neither the selection. We are awaiting the appreciation of this manuscript so that we get the approval, thus, after that we can start the review.

I am sorry that we cannot be more positive on this occasion, but we hope that you appreciate the reasons for this decision.

LETTER IN RESPONSE TO EDITORS

Yours sincerely,
Marianne Clemence, DPhil
Associate Editor
PLOS ONE

Dear Dr. Lopes-Junior,
Thank you for contacting us about your submission.
I’ve passed your query to our editorial team, who will reassess your manuscript in light of the comments you shared with me.
Please do not hesitate to contact us again if you have any questions, or if you do not hear back from us within several weeks.
With kind regards
Kate Massingham
Editorial Office
PLOS | plos.org

Response: Thank you so much for your reply. I am looking forward to hearing from the Editorial Board soon.

PONE-D-21-24128
School reopening and risks accelerating the COVID-19 pandemic: a systematic review and meta-analysis protocol
PLOS ONE

Dear Dr. Lopes-Junior,
I am writing to you about your appeal on the editorial decision for your submission to PLOS ONE above.
As you are aware, manuscripts submitted to PLOS ONE are assessed based on the journal’s publication criteria. At present, the manuscript does not meet our criterion for publication requiring that the article be presented in an intelligible fashion and written in standard English.

If you decide to resubmit your manuscript, please ensure that you undertake revisions to carefully address all of the items described in your original decision letter. Please include the original manuscript number in your cover letter and provide a summary of the revisions undertaken since the original submission. We will carefully review a resubmitted manuscript, so please ensure that your revision is thorough.

Response: Regarding the quality of the English, as per recommended we have paid for having our manuscript copy edited for language usage and grammar, by a third party or a professional service in order to become adequate standard for peer review (we have uploaded the certificate in the PLOS One manager system).

Please note that we cannot advise on the outcome of any resubmission based on your attachment. However, you may wish to undertake further revisions to ensure that the quality of the language is at an adequate standard for peer review.

Thank you for your interest in PLOS ONE.

Response: Thank you so much for this new opportunity for present our manuscript.
Best wishes,
Marianne Clemence, DPhil
Staff Editor
PLOS ONE

The authors certify that we have no funding or conflicts of interest to disclose. In addition, all authors have participated in the concept and design, analysis and interpretation of data, drafting or revising of the manuscript, and have approved the manuscript as submitted. Also, this is an original study that has not been published previously and is not under consideration for publication in another journal. We believe it will be of great interest to your readers.

Yours Sincerely,
The authors
