# Supplementary material for: School reopening and risks accelerating the COVID-19 pandemic: A systematic review and meta-analysis protocol
Source: PLoS One. 2021 Nov 17;16(11):e0260189. doi: 10.1371/journal.pone.0260189 (PMC8598030; doi:10.1371/journal.pone.0260189)
Supplement: Attachment — Submitted filename: Response to Reviewers.docx [file pone.0260189.s004.docx]

**LETTER IN RESPONSE TO REVIEWERS**

Vitória-ES, Brazil, October 2, 2021

Dear Editorial Board of the *PLoS One*

We are resubmitting our Manuscript ID: PONE-D-21-25744, entitled “School reopening and risks accelerating the COVID-19 pandemic: a systematic review and meta-analysis protocol”. We sincerely appreciate your valuable feedback and would like to thank you for all suggestions and comments. The editorial as well as the reviewers request was addressed and summarized below with changes in the main text. All co-authors have agreed to the resubmission after these revisions.

Below we have responded in detail point by point the items raised by reviewers, which have all been addressed.

Muhammad Shahzad Aslam, Ph.D.,M.Phil., Pharm-D
Academic Editor - PLOS ONE

Journal Requirements:

When submitting your revision, we need you to address these additional requirements.

1. Please ensure that your manuscript meets PLOS ONE's style requirements, including those for file naming. The PLOS ONE style templates can be found at
   [https://journals.plos.org/plosone/s/file?id=wjVg/PLOSOne_formatting_sample_main_body.pdf](https://journals.plos.org/plosone/s/file?id=wjVg/PLOSOne_formatting_sample_main_body.pdf) and 
   [https://journals.plos.org/plosone/s/file?id=ba62/PLOSOne_formatting_sample_title_authors_affiliations.pdf](https://journals.plos.org/plosone/s/file?id=ba62/PLOSOne_formatting_sample_title_authors_affiliations.pdf)

**Response:** Ok. Done.

2. Please review your reference list to ensure that it is complete and correct. If you have cited papers that have been retracted, please include the rationale for doing so in the manuscript text, or remove these references and replace them with relevant current references. Any changes to the reference list should be mentioned in the rebuttal letter that accompanies your revised manuscript. If you need to cite a retracted article, indicate the article’s retracted status in the References list and also include a citation and full reference for the retraction notice.

**Response:** Ok. Done.

3. In your Data Availability statement, you have not specified where the minimal data set underlying the results described in your manuscript can be found. PLOS defines a study's minimal data set as the underlying data used to reach the conclusions drawn in the manuscript and any additional data required to replicate the reported study findings in their entirety. All PLOS journals require that the minimal data set be made fully available. For more information about our data policy, please see [http://journals.plos.org/plosone/s/data-availability](http://journals.plos.org/plosone/s/data-availability).

Upon re-submitting your revised manuscript, please upload your study’s minimal underlying data set as either Supporting Information files or to a stable, public repository and include the relevant URLs, DOIs, or accession numbers within your revised cover letter. For a list of acceptable repositories, please see[http://journals.plos.org/plosone/s/data-availability#loc-recommended-repositories](http://journals.plos.org/plosone/s/data-availability#loc-recommended-repositories). Any potentially identifying patient information must be fully anonymized.

**Response:** Ok. Done.

Important: If there are ethical or legal restrictions to sharing your data publicly, please explain these restrictions in detail. Please see our guidelines for more information on what we consider unacceptable restrictions to publicly sharing data: [http://journals.plos.org/plosone/s/data-availability#loc-unacceptable-data-access-restrictions](http://journals.plos.org/plosone/s/data-availability#loc-unacceptable-data-access-restrictions). Note that it is not acceptable for the authors to be the sole named individuals responsible for ensuring data access.

We will update your Data Availability statement to reflect the information you provide in your cover letter.

[Note: HTML markup is below. Please do not edit.]

**Reviewers' comments:**

**Reviewer's Responses to Questions**

**Comments to the Author**

1. Does the manuscript provide a valid rationale for the proposed study, with clearly identified and justified research questions?

The research question outlined is expected to address a valid academic problem or topic and contribute to the base of knowledge in the field.

Reviewer #1: Yes

Reviewer #2: Yes

2. Is the protocol technically sound and planned in a manner that will lead to a meaningful outcome and allow testing the stated hypotheses?

The manuscript should describe the methods in sufficient detail to prevent undisclosed flexibility in the experimental procedure or analysis pipeline, including sufficient outcome-neutral conditions (e.g. necessary controls, absence of floor or ceiling effects) to test the proposed hypotheses and a statistical power analysis where applicable. As there may be aspects of the methodology and analysis which can only be refined once the work is undertaken, authors should outline potential assumptions and explicitly describe what aspects of the proposed analyses, if any, are exploratory.

Reviewer #1: Yes

Reviewer #2: Yes

3. Is the methodology feasible and described in sufficient detail to allow the work to be replicable?


Descriptions of methods and materials in the protocol should be reported in sufficient detail for another researcher to reproduce all experiments and analyses. The protocol should describe the appropriate controls, sample size calculations, and replication needed to ensure that the data are robust and reproducible.

Reviewer #1: Yes

Reviewer #2: Yes

4. Have the authors described where all data underlying the findings will be made available when the study is complete?

The [PLOS Data policy](https://journals.plos.org/plosone/s/materials-and-software-sharing) requires authors to make all data underlying the findings described in their manuscript fully available without restriction, with rare exception, at the time of publication. The data should be provided as part of the manuscript or its supporting information, or deposited to a public repository. For example, in addition to summary statistics, the data points behind means, medians and variance measures should be available. If there are restrictions on publicly sharing data—e.g. participant privacy or use of data from a third party—those must be specified.

Reviewer #1: Yes

Reviewer #2: Yes

5. Is the manuscript presented in an intelligible fashion and written in standard English?

PLOS ONE does not copyedit accepted manuscripts, so the language in submitted articles must be clear, correct, and unambiguous. Any typographical or grammatical errors should be corrected at revision, so please note any specific errors here.

Reviewer #1: Yes

Reviewer #2: No

6. Review Comments to the Author

Please use the space provided to explain your answers to the questions above and, if applicable, provide comments about issues authors must address before this protocol can be accepted for publication. You may also include additional comments for the author, including concerns about research or publication ethics.

You may also provide optional suggestions and comments to authors that they might find helpful in planning their study.

**Reviewer #1:**

Interesting and timely analysis.
**Response:** Thank you so much for your comments as well as suggestions.

Some issues

- I'm not sure that authors can use fixed effect with a meta-analysis of observational studies

**Response:** You are right. We made adjustments in the data synthesis and into abstract. Thank you very much.

In fact, random effects models that assume that the effect of interest is not the same across studies. In this sense, they consider that the studies that are part of the meta-analysis form a random sample of a hypothetical population of studies. For this reason, they create combined results with a greater confidence interval, which is why they are the most recommended. However, despite having this advantage, methods with random effects are criticized for giving greater weight to smaller studies (Egger et al., 2001).

Egger, M.; Smith, G.D. & Altman, D.G. (Ed.s) (2001). Systematic reviews in health care: Meta-analysis in context. 2nd ed. London: BMJ Books.

- Methods: school and pre school are different managed. For example face masks in some countries are compulsory for school but not pre school
**Response:** Thank you for raising this timely issue. Actually, the results of this systematic review also will be presented stratifying them by level of education. We have add this on data synthesis. Thanks!

Methods IF should not be included, actually it does not always reflect high quality paper

**Response:** OK. We agree with you. We have removed the IF from the data extraction form as per suggested.

**Reviewer #2**:

Congratulation to the authors. This is a great contribution to science, particularly in the COVID-19 pandemia.

**Response:** Thank you so much for your comments as well as suggestions.

Some few suggestions and comments:
Review the standard english, particularly in methods section.

**Response:** Ok. Done. The new version of this manuscript was edited for proper English language, grammar, punctuation, spelling, and overall style by two of the highly qualified native English speaking, as recommended by you. (Please, see attached the revision certificate).

Put the extract form of information in the supplementary material;

**Response:** Ok. Done.

In the manuscript, authors pointed that they will take into account the degree of vaccination. How this topic would be taken? Some explanation will be great.

**Response:** Thank you very much for this interesting question. We reflected on this issue and decided to withdraw this analysis, once, it will be difficult to obtain data from each study regarding vaccination (comprising the period of primary data collection considering this endpoint). Instead, we thought of addressing the protective factor "vaccination" in the discussion section of our systematic review.

Why authors will search all database without date restrictions, since the COVID-19 pandemic started in 2019?

**Response:** Because according to the Cochrane Collaboration Handbook (Higgins et al., 2021), dates should not be restricted in systematic reviews. Although, we will obviously only retrieve articles from the end of 2019 onwards.

Higgins JPT, Thomas J, Chandler J, Cumpston M, Li T, Page MJ, Welch VA (editors). Cochrane Handbook for Systematic Reviews of Interventions version 6.2 (updated February 2021). Cochrane, 2021.

The authors will take into account the mandatory or not use of mask in schools? This topic did not appear in the protocol.

**Response:** Thank you for your valuable comments. We have added this issue in data synthesis. Yes, we will take into account whether or not masks are mandatory in schools, as well as other measures to mitigate the spread of the virus. Based on the outcomes of the studies included in the review, we will perform subgroup analyses.

The authors certify that we have no funding or conflicts of interest to disclose. The manuscript is consistent with the Guidelines for Authors of the PLOS One. Also, this is an original study that has not been published previously and is not under consideration for publication in another journal.

Yours Sincerely,

The authors
